# Does Migration Affect a Child’s Immunization Coverage? A Cross-Sectional Analytical Study of India

**DOI:** 10.3390/vaccines14090744

**Published:** 2026-08-27

**Authors:** Pawan Kumar, Pritu Dhalaria, Sanjay Kapur, Ajay Kumar Verma, Ajeet Kumar Singh, Pretty Priyadarshini, Kapil Singh, Bhupendra Tripathi, Arindam Ray

**Affiliations:** 1 Immunization Division, Ministry of Health & Family Welfare, New Delhi 110011, India; 2 Immunization Technical Support Unit, Ministry of Health & Family Welfare, Government of India, New Delhi 110070, India; 3 John Snow India, New Delhi 110070, India; 4 Gates Foundation, New Delhi 110067, India

**Keywords:** parental migration, immunization coverage, determinants, intervention, IA2030

## Abstract

**Introduction**: India’s immunization initiatives are among the largest globally, characterized by a substantial birth cohort of 27 million children annually, and have achieved significant progress in increasing coverage through the UIP. However, there are still challenges that persist, and multiple determinants contribute to the existing challenges; migration is one of them. Migration has always been a key driver of socio-economic and demographic changes, particularly in low and middle-income countries (LMICs). Specifically, there is a need to better understand the vulnerabilities of immunization among recent migrants. To examine this, the study explores the association between a mother’s recent migration and the full immunization coverage of children aged 12–23 months in India. **Data & Methods**: Our study utilized data from the National Family Health Survey-5 (2019–21). The outcome variable of interest in this study is the full immunization among children aged 12–23 months (receipt of all basic vaccinations). The primary predictor variable in this study was recent maternal migration, defined as the mother’s duration of residence at the current place of residence. We used a series of multivariate logistic regression models to examine the relationship between full Immunization among children and maternal migration status, with some data restrictions in the models. **Results**: The results show a 17 percentage-point difference in full immunization between children of migrant and non-migrant mothers. The adjusted odds ratios of children of mothers who had recently migrated had significantly lower odds of full immunization (aOR: 0.39, 95% CI: 0.35–0.43) compared to children whose mothers had not recently migrated. Even across the household wealth quintile and social groups, the recent migration of the mother was associated with being less likely to be fully immunized among children 12–23 months. **Conclusions**: The findings of this study provide significant quantitative evidence that recent migration (less than 3 years) of mother is a key factor influencing Immunization coverage and is a predictor of full immunization among children aged 12–23 months in India. The recent migration was consistently linked with lower odds of full immunization coverage across different household wealth levels and social groups. This study suggests that children of recently migrated mother are a vulnerable subgroup of the population at risk of not receiving full immunization among children aged 12–23 months.

## 1. Introduction

The Immunization Agenda (IA) 2030 promotes equitable access to full immunization for all, irrespective of location, age, gender, or socioeconomic barriers [1]. It recognizes vaccinations as the most effective public health interventions to safeguard children’s health against disease. Vaccinations advance global health equity by reducing inequalities between high-income and socioeconomically disadvantaged communities [2,3]. Since the introduction of the Expanded Programme on Immunization (EPI) in 1974, vaccinations have prevented 154 million deaths worldwide, including 146 million deaths among children under five, with 101 million of these deaths occurring among children less than one year old. These childhood survival gains have occurred across all WHO regions [4]. Estimates suggest that childhood vaccination programs in low- and middle-income countries (LMICs) for ten pathogens from 2000 to 2030 will prevent approximately 69 million deaths. Of these, 37 million deaths were avoided between 2000 and 2019, compared to scenarios without vaccination. Improved worldwide coverage and new vaccine developments could reduce life-time mortality for the 2019 birth cohort by up to 72% [5].

India’s immunization initiatives are among the largest globally, catering to the world’s largest birth cohort of approximately 26 million children and 29 million pregnant women annually [6]. The full immunization coverage (FIC) has improved over recent decades, from 35% in 1992–93 to 76% in 2019–21 [7,8]. With 76.4% FIC and 20% of children partially immunized, India is on track to achieve the global target of the Immunization Agenda 2030 (IA 2030) [9]. Despite India’s efficient immunization system, a significant number of children remain under vaccinated. Coverage drops have been observed across all multidose childhood vaccines, highlighting the persistent challenge of vaccine completion beyond the first dose and substantial gaps and inequalities across regions and socioeconomic groups [10]. Factors like socioeconomic status, maternal education, limited awareness, vaccine hesitancy, and gender bias are well recognized contributors to incomplete vaccination coverage in India. Additional challenges, such as difficult terrain and strained health infrastructure, further compound the issue [7,9,11,12,13].

Among the various reasons for partial or non-immunization, migration stands out as a significant barrier to immunization uptake. Migrant populations represent some of the most vulnerable groups that face adverse health outcomes and barriers to healthcare services, including childhood immunization [14]. Suboptimal vaccination coverage is a significant concern for migrant populations globally, with implications for both individual and public health. In European countries, evidence suggests that vaccination coverage among both migrant adults and children is markedly lower, around 50%, compared to the native population [15]. Consistently lower coverage in migrant groups is not merely an individual health issue. It is also linked to vaccine-preventable disease (VPD) outbreaks, including measles, varicella, mumps, rubella, and hepatitis A [16]. Similar patterns are observed in the Middle East and North Africa (MENA) region. Data from 15 countries in this region show that routine childhood vaccination coverage can be as low as 36% in certain countries, such as Lebanon, Morocco, and Sudan [17]. Specifically in India, studies from certain regions have also documented low full immunization coverage among children of migrant construction workers, ranging from 26% to 30% [18,19]. According to the recent Periodic Labour Force Survey (PLFS) 2020–21 estimates, India’s migration rate is approximately 30%. This rate shows a variation between regions, with 26.5% in rural areas and 34.9% in urban areas [20]. This significant demographic shift, in turn, leads many people to establish new living arrangements in urban slums and peri-urban areas. This transition impacts access and utilization of health services, including routine immunization of infants and children. Achieving full immunization coverage is a continuous, year-long process that requires multiple visits, as outlined in the National Immunization Schedule. Therefore, migration during this critical period can lead to dropouts and delays in immunization uptake.

Migration serves as a significant catalyst for socio-economic and demographic shifts, particularly in LMICs. The association between migration and health outcomes is diverse and is influenced by several factors. These include the characteristics of the migrant, the stage of life at which the migration occurs, the specific origins and destinations involved, and the underlying motivations for migrating [14,21,22].

### 1.1. Conceptual Framework

Access to healthcare services is a major concern in social science and public health research for vulnerable populations. The utilization of health services, including immunization, by migrants is commonly analyzed through three theoretical perspectives: migrant disruption, adaptation, and assimilation [14,23,24]. The disruption perspective holds that migration disrupts the natural progression of demographic event s in migrants’ lives, thereby impacting health behaviors and outcomes. Disruption is assumed to bring short-term behavioral changes. This is often due to insecurity in livelihoods, unmet basic needs, and vulnerabilities in existing health beliefs, practices, and social norms. Conversely, the adaptation hypothesis proposes that migrants intentionally, although temporarily, modify their behavior to conform to the prevailing social norms of their new environment. The assimilation hypothesis suggests that migrants progressively internalize the norms of the host society before fully integrating into the community [25,26,27,28].

**Figure 1:**
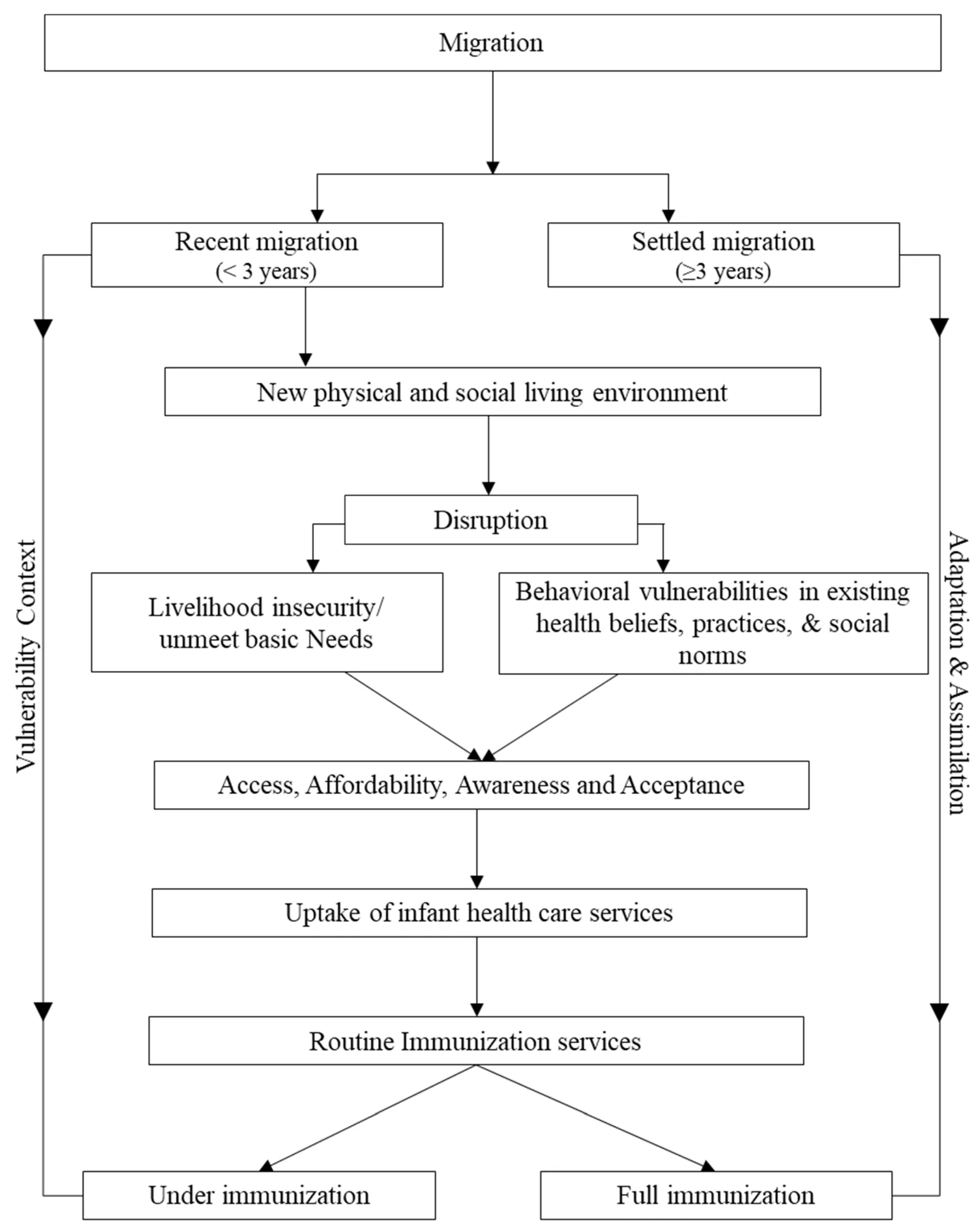
Hypothesized theoretical framework of how migration influences immunization coverage among children.

The literature identifies several dimensions related to the root causes of vaccination coverage gaps and vaccine uptake, often categorized into key themes. The evidence-informed 4As—Access, Affordability, Awareness, and Acceptance. Taxonomy is a prominent theoretical framework for determining vaccine uptake behavior in childhood and adults [29]. This demonstrates that factors such as livelihood insecurity, health beliefs, behavioral vulnerability, and social norms collectively influence vaccination access and outcomes. This complex interplay directly influences the consistent uptake of routine vaccines and their coverage. Language, literacy, and communication barriers emerged as critical factors that can reduce vaccine uptake [30]. Studies indicate that migrants often face challenges accessing public health services, including child vaccination. Beyond financial access concerns, non-financial barriers also impede vaccination, even among caregivers who intend to immunize their children. These included opportunity costs, competing priorities, and rigid session timings and schedules [29,31]. Information and perception-related issues contribute to hesitancy, often stemming from limited exposure to information and the availability of a physician or frontline health worker. Thus, the 4As taxonomy helps classify and describe different potential causes of lower vaccination uptake amongst migrants. Building on this framework, our study hypothesizes that recent migrants may be less likely than settled migrants and the native population to complete their full immunization (Figure 1). The hypothesis has been translated into a flow diagram to better portray the effects of migration on vaccine uptake.

### 1.2. Literature Review

Understanding the immunization status of migrant children is challenging due to the complex nature of migration. There are very few studies in India that have examined the relationship between migration and children’s immunization status. Existing nationally representative studies show inconsistent findings regarding the association between migration status and full immunization coverage. Notably, no studies have specifically analyzed the relationship between short-term migration (<3 years) and full immunization for children aged 12–23 months using a nationally representative sample. Two studies on migration and childhood immunization exist, but both have statistical and conceptual limitations. Prusty and Keshri (2015) investigated the association between child nutrition and immunization among migrants and non-migrants using urban sample data from the National Family Health Survey (NFHS) 2005–06 [32]. Mishra et al. (2020) examined migration across socioeconomic groups and immunization coverage using NFHS 2015–16 data [33]. Their findings suggested that migrated children (<4 years) had improved vaccination coverage and more likely to take the vaccine compared to non-migrants. Some state and city-specific studies indicate that immunization coverage among migrant children was either found to be low or no significant difference compared to non-migrants and settled migrants, depending on the location [19,34,35]. Conceptually, these studies did not clearly distinguish recent migrants from other groups, often treating all periods of residence as migration. However, these studies only fitted bivariate associations and failed to established relationship between migration status and immunization among children, along with covariates.

Recent migrants are often primarily focused on meeting their daily livelihood needs and tend to remain socially isolated compared to settled migrants and native populations. Unlike long-term residents, new arrivals often lack established local support networks to help them navigate the healthcare system or access information about available services. This challenge is further compounded by poor living and housing conditions and limited access to essential healthcare. In line with the goals of Immunization Agenda 2030 (IA2030) and its commitment to “leave no child behind,” comprehensive tracking of children is essential; however, migrant children remain one of the most difficult groups to reach across the stratum.

Migration is a major and growing phenomenon in developing countries such as India, yet its implications for child health, particularly immunization, remain insufficiently understood. Our study aims to examine the relationship between recent parental migration and children’s complete immunization status among those aged 12–23 months using the most recent nationally representative data. There is a clear gap in the literature regarding the nuanced stratification of migrant populations, especially in distinguishing between recent and settled migrants and non-migrant groups, and in assessing differences in immunization coverage across these categories. The duration of stay is a critical but often overlooked dimension of migration dynamics, as recent migrants are likely to be the most vulnerable and have lower uptake of public health services, including child immunization, compared to other groups. By addressing this gap, the study seeks to generate deeper insights into the specific vulnerabilities migrant populations face in accessing childhood immunization services. These findings can help identify high-risk districts and clusters with significant migrant populations, enabling more targeted, context-sensitive interventions. Such evidence is essential for strengthening programmatic strategies and accelerating progress toward equitable immunization coverage in line with IA2030 goals.

## 2. Methodology

### 2.1. Data Sources

**Figure 2:**
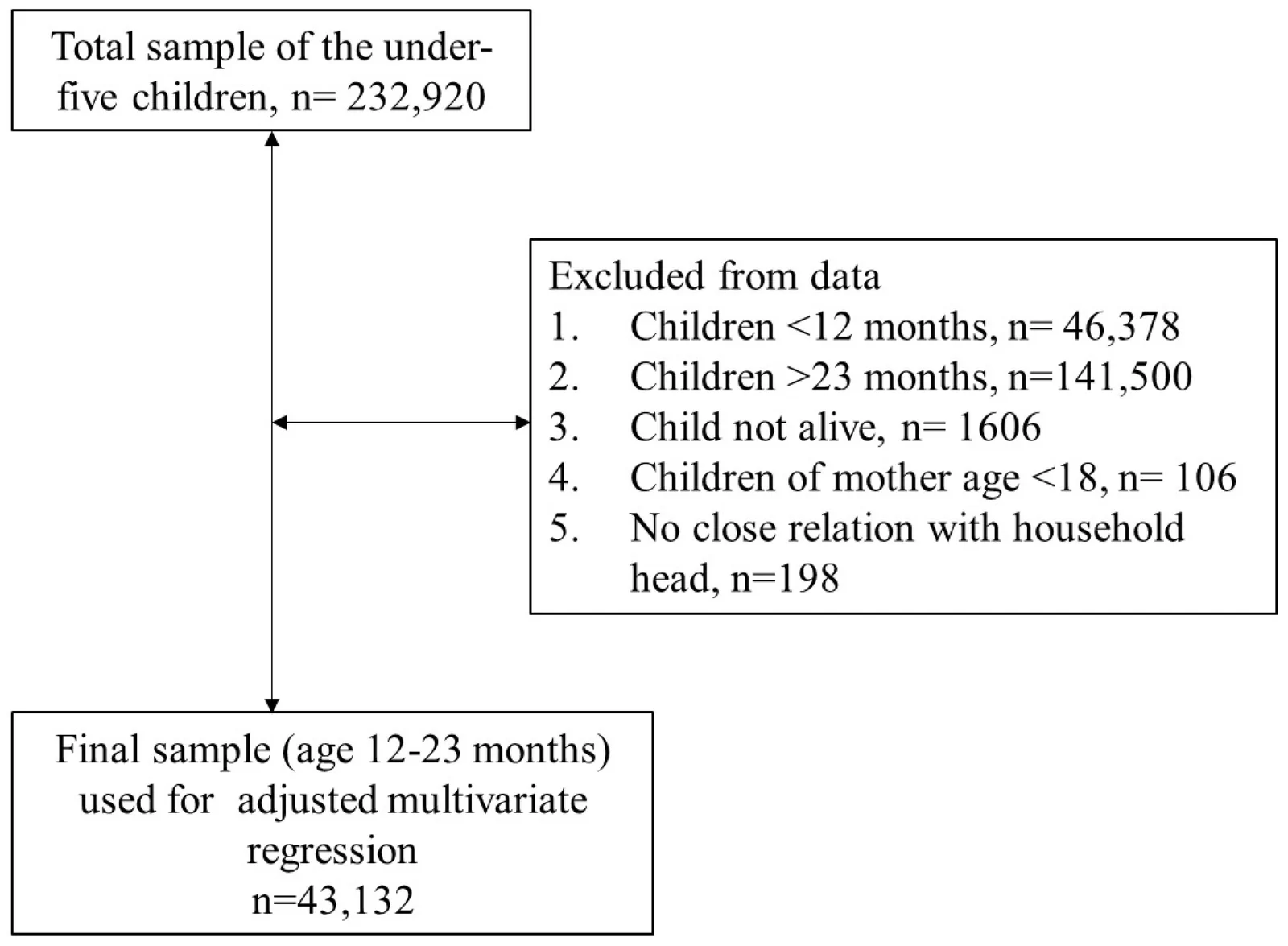
Flow chart of the analytical sample, NFHS 2019–2021.

The study is based on data from the National Family Health Survey (NFHS-5) 2019–2021. NFHS-5 is a nationally representative survey conducted under the Ministry of Health and Family Welfare (MoHFW), Government of India. The primary objective of NFHS 2019–2021 was to collect high-quality data on health and family welfare indicators, enabling policymakers to track progress in India’s health sector and identify gaps in specific health programs. The protocol for the NFHS-5 survey, including the content of all survey questionnaires, was approved by the IIPS Institutional Review Board and the ICF Institutional Review Board. The protocol was also reviewed by the U.S. Centers for Disease Control and Prevention (CDC). The NFHS data provides information on maternal and child health, including fertility, infant and child mortality, maternal health, and uptake of healthcare services. The sample size of NFHS-5 was designed to generate indicators at district, state, and union territory (UT) levels, covering 707 districts, 28 states, and 8 UTs. NFHS-5 employed a stratified two-stage sampling method to ensure representativeness, operational feasibility, and cost-efficiency. In the first stage, the Indian Census provided the sampling frame for selecting primary sampling units (PSUs) in rural areas and census enumeration blocks (CEBs) in urban areas. In the second stage, 22 PSUs and CEBs were randomly selected in each district. 724,115 women aged 15–49 from 636,699 chosen households were interviewed. NFHS-5 collected information on child health and services from a sample of 232,920. Accordingly, according to the full immunization coverage definition, we excluded children who had died, children above 23 months, and children less than 12 months. We further excluded children whose mothers were younger than 18 years to ensure a consistent maternal age eligibility criterion and children whose mothers did not have a close relationship to the household head. Consequently, we included 43,132 children aged 12–23 months to meet our study goals (Figure 2).

### 2.2. Outcome Variable

The outcome variable of interest in this study is the coverage of full immunization among children 12–23 months old. A child aged 12–23 months is considered fully immunized when they have received a dose of BCG, three doses each of DPT and Polio, and one dose of measles vaccine, as documented on the vaccination card or by direct reporting by the mother. Around 87% of immunization history was recorded from the vaccination card, and the remaining (13%) from maternal recall. These vaccines are administered in accordance with India’s national immunization schedule (NIS). Under this schedule, BCG is given at birth, the first, second, and third doses of DPT and Polio are administered at 6, 10, and 14 weeks of age, respectively, and the measles vaccine is administered between 9 and 11 months of age.

### 2.3. Predictor Variable

Predictor variables were chosen because they have been identified as important covariates of child immunization in prior studies. The primary predictor variable in this study is migration durational status. The disruption hypothesis is associated with migration duration, which assumes that recent migrants have lower utilization of health care services, compared with non-recent migrants. NFHS-5 does not provide direct information on the respondents’ migration status to their previous place of residence. Indeed, NFHS-5 inquires about how long the respondents, the children’s mothers, have been residing at their current address, offering three answer choices: ‘years in numbers’, ‘always’, and ‘visitors’. We classified recent migration of the mother as living at the current residence for less than 3 years, including visitors with a relationship to the household, such as a daughter, daughter-in-law, or grandchild. It is assumed that these movements result in minimal alteration to the physical or social environment concerning healthcare access and behavior. Our three-year threshold for ‘recent migration’ covers both the recommended vaccination window (birth to 11 months) and the period used to assess full immunization coverage (12–23 months). In addition, this period overlaps with important antenatal, delivery, postnatal, and early childhood health-care services, during which relocation may disrupt continuity of care and access to scheduled immunization services. Close relatives (visitors) of the household are also included in the scope of temporary childbirth migration. This is because they benefit from additional care and support, are expected to have reduced workload at the natal home, and experience lower health care related costs.

We included the child and maternal socio-demographic background variables such as gender of child, mother’s age at childbirth (15–19 years, 20–29 years and 30+ years), maternal education in years (no schooling, <5 years complete, 5–8 years complete, 9–12 years, and >12 years), media exposure at least once in a week (No, Yes), child birth order (1st order, 2nd, 3rd, 4th & more order), institutional delivery (Public health facility, Private health facility, No health facility), Intended birth (Yes, No), current place of residence (Urban and Rural), wealth quintile (Poorest, Poorer, Middle, Richer, and Richest), religion (Hindu, Muslim, Christian, Sikh and Others), regions of India (North, Central, East, Northeast, West and South). The caste of the children was classified into four social groups: Scheduled Caste (SC), Scheduled Tribe (ST), Other Backward Class (OBC), and Others.

### 2.4. Statistical Analysis

Initially, our study analyzed the distribution of the sample in relation to the outcome and predictor variables, along with various socio-demographic factors (Table 1). Next, we estimated the inequality in full immunization by recent migration status across 36 Indian states/UTs, along with several socio-economic variables, including wealth quintile, maternal education, and social groups. We found that the mean VIF was 1.70, with individual VIFs ranging from 1.00 to 3.05, indicating no evidence of problematic multicollinearity among the covariates. We used a series of multivariate logistic regression models to examine the relationship between full immunization and recent migration (Yes/No) (Table 2). By implementing this methodology, we were able to perform robustness and sensitivity analysis of our framework using three sequential specifications. **Model I** utilized the full analytic sample of 43,132 children aged 12–23 months. **Model II** was restricted to children whose mothers were currently living with their husbands/partners and whose husbands/partners had no other wives, resulting in an analytic sample of 37,172 children. **Model III** applied the same restrictions as Model II and additionally excluded temporary visitors to the current place of residence by treating them as missing in the recent migration variable, resulting in a final analytic sample of 36,357 children. Furthermore, we created two additional supplementary models (Tables 3 and 4) of recent migration, one for migration of less than 3 years and permanent residence, and another for migration of less than 3 years and settled migration (migration of 3 years or more), to examine the relationship between full immunization and recent migration as a sensitivity analysis. Finally, we estimated a series of multivariable logistic regression models stratified by household wealth quintile and social group to examine whether the association between recent maternal migration and full immunization varied across socioeconomic groups. All results from multivariate logistic regression models were reported as adjusted odds ratios (aOR) with 95% confidence intervals (CI). Statistical significance was set at *p* < 0.05, *p* < 0.01, and *p* < 0.001. All analyses considered the sampling design and sampling weights and were performed with Stata 19 software. As this study did not aim to establish causal relationships, the estimates should be interpreted as measures of association rather than causal effects. The study was conducted and reported in accordance with STROBE guidelines for observational cohort studies.

## 3. Results

The distribution of the study sample is shown in Table 1. The table indicates that 76.6% of the children were fully immunized, while 23.4% reported being under-immunized, including both partially immunized children (dropout) and zero-dose children. Additionally, 9.6% of the sample reported recent migration within the past three years, including those who were visitors at their current residence, while 90.4% were classified as non-migrant, having permanently settled in their current residence.

**Figure 3:**
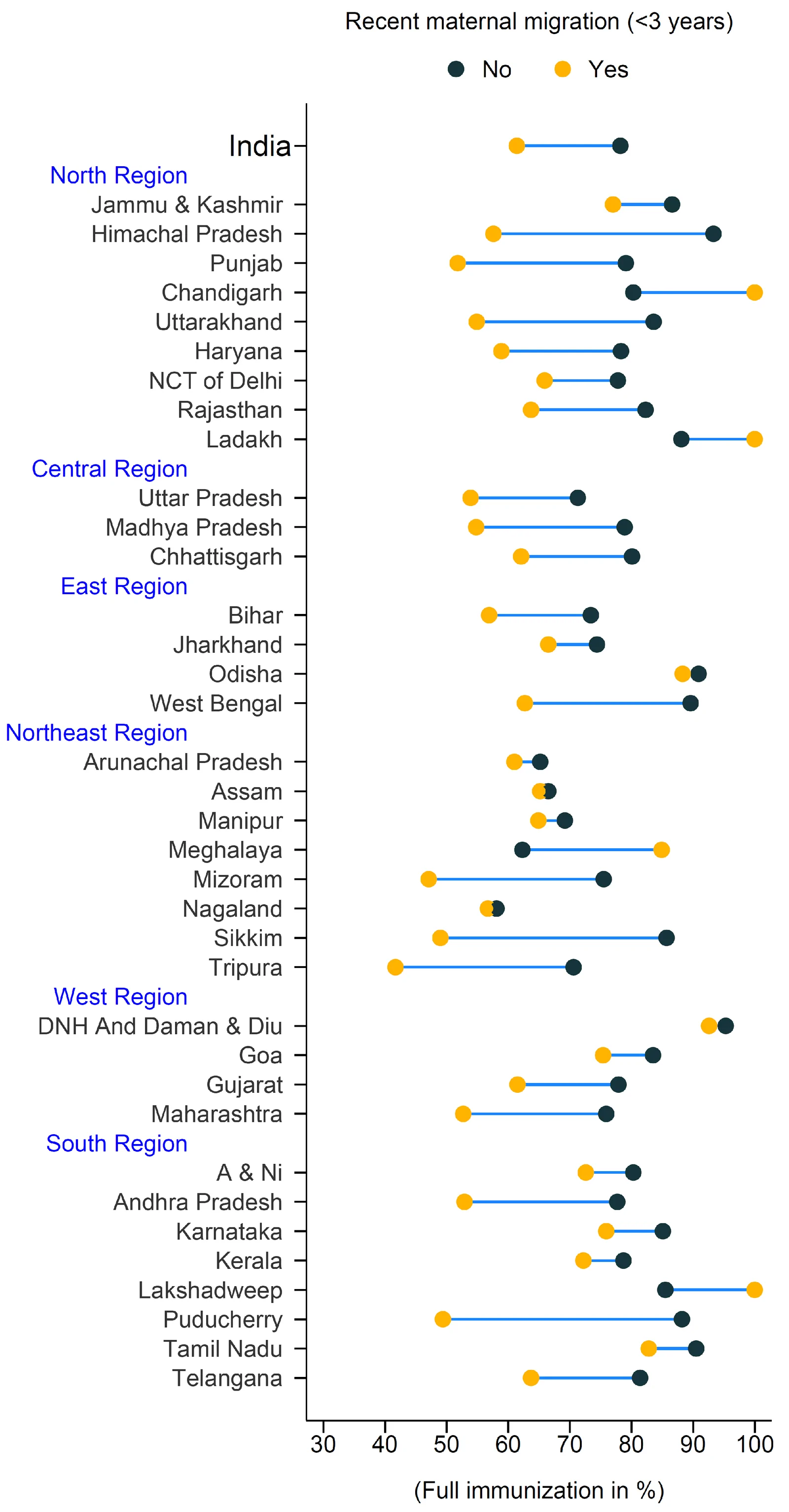
Inequality in full immunization among children aged 12–23 months by recent maternal migration status across States/UTs, India, 2019–2021.

**Figure 4:**
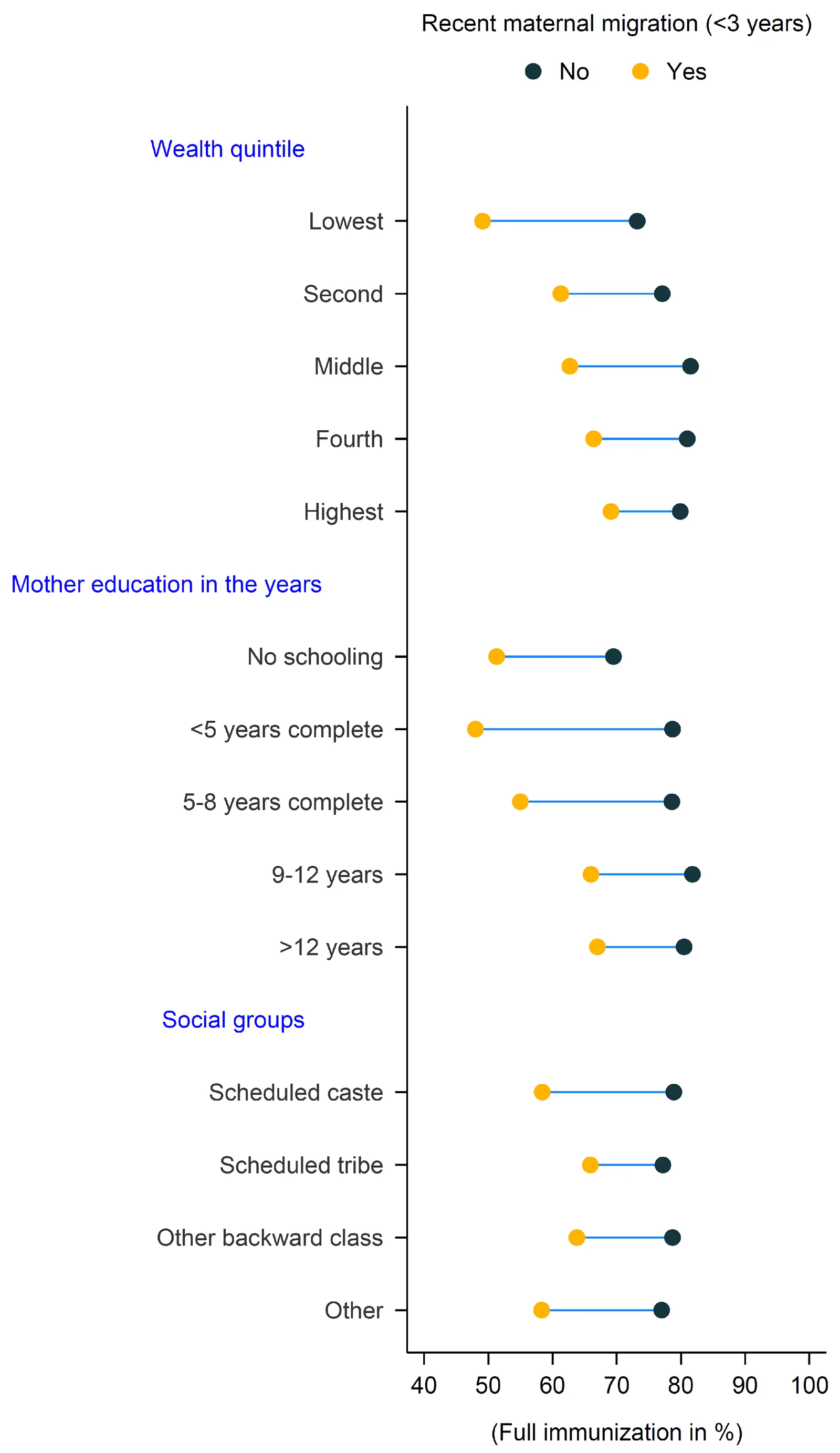
Inequality in full immunization among children aged 12–23 months by recent maternal migration status across different socio-economic backgrounds, mothers’ education status, and social groups in India, 2019–2021.

The study shows disparities in full immunization coverage across different states/UTs in India and among various socio-economic groups, particularly in relation to recent maternal migration. A notable 17 percentage point disparity in full immunization rates among children is observed when comparing those with recent migration status of their mother, as shown in Figure 3. Among major states, only Odisha (3%) and Assam (1%) showed negligible differences in full immunization coverage between children of recent migrants and non-migrants mothers. Conversely, Ladakh, Chandigarh, Meghalaya, and Lakshadweep showed lower immunization rates among children of non-recent migrants than among those of recent migrants. Figure 4 displays full immunization coverage among children from different socio-economic backgrounds, categorized by their mothers recent migration status. The data indicate that children from the poorest households, those whose mothers have less than five years of education, and who are from the Scheduled Castes, have lower immunization rates, especially among those who are children of recent maternal migrants.

Table 2 shows the results of a multivariate logistic regression analysis on full immunization among children aged 12–23 months. In Model I, the odds of full immunization were lower among children whose mothers had recently migrated (aOR: 0.39, 95% CI: 0.35–0.43) than among children whose mothers had not recently migrated. In Model II (restricted to children whose mothers were currently living with their husbands/partners and whose husbands/partners had no other wives), the odds ratio for full immunization was lower among children of recently migrated mothers (aOR: 0.38, 95% CI: 0.34–0.44) than children of non-migrated mothers. Furthermore, Model-III applied the same restrictions as Model-II and additionally excluded temporary visitors to the current place of residence. In this model, children of recently migrated mothers also had lower odds of full immunization than children of non-migrant mothers (aOR: 0.52, 95% CI: 0.45–0.62). This pattern suggests consistently lower odds of full immunization among children of recently migrated mother across different analytical models.

Table 3 shows the results of a multivariate logistic regression analysis on full immunization among children aged 12–23 months in relation to maternal recent migration and permanent residence at their current residential place. The analysis found that children whose mothers had recently migrated (less than 3 years ago) had lower odds of being fully immunized than children whose mothers were permanent residents (aOR: 0.67, 95% CI: 0.54–0.83). We further restricted to children whose mothers were currently living with their husbands/partners and whose husbands/partners had no other wives (Model II of Table 3). The findings were consistent, as children of recently migrated mothers had lower odds of full immunization (aOR: 0.70, 95% CI: 0.53–0.92) compared to children whose mother had always resided at their current location.

Table 4 presents an association result of full immunization in relation to recent migration and settled migration (living at the current location for 3 or more years but not permanently residing there) among children aged 12–23 months. In Model I, the odds ratio for full immunization was lower among children of recently migrated mothers (OR: 0.52, 95% CI: 0.46–0.60) compared with children of settled migrant mothers. In Model-II, restricted to children whose mothers were currently living with their husbands/partners and whose husbands/partners had no other wives, the odds ratio for full immunization was lower (OR: 0.56, 95% CI: 0.48–0.66) among children of recently migrated mothers compared with children of settled migrant mothers.

Table 5 presents the results of a multivariate logistic regression examining full immunization among children aged 12–23 months with maternal migrant status, stratified by household wealth quintile. The analysis reveals that children of recently migrated mothers across all household wealth quintiles had significantly lower odds of being fully immunized than their non-migrant counterparts. Specifically, the odds ratios (aOR) were as follows: poorest (aOR: 0.29, 95% CI: 0.24−0.36), poor (aOR: 0.43, 95% CI: 0.35−0.53), middle (aOR: 0.34, 95% CI: 0.26−0.43), richer (aOR: 0.42, 95% CI: 0.33−0.53), and richest (aOR: 0.52, 95% CI: 0.40−0.67). This shows that the association was consistently negative across all wealth quintiles, suggesting that the association between recent maternal migration and lower odds of full immunization was not limited to a particular socioeconomic group.

Table 6 presents the relationship between full immunization among children aged 12–23 months and maternal migration status, stratified by social group. The results indicate lower odds of full immunization among children in all social groups whose mothers have recently migrated. The odds ratios were as follows: Schedule Caste (aOR: 0.35, 95% CI: 0.28−0.42), Schedule Tribe (aOR: 0.48, 95% CI: 0.34−0.69), Other Backward Classes (aOR: 0.40, 95% CI: 0.35−0.46), and Others (aOR: 0.38, 95% CI: 0.30−0.48) compared with their non-migrant counterparts.

## 4. Discussion

This study investigated the relationship between maternal migration and full immunization coverage among their children aged 12–23 months in India. Recent maternal migration was defined as the mother residing in the current location for less than 3 years. The study revealed five significant findings regarding the full immunization of children and migration status of mothers in India. First, 9.6% of children lived with their mothers at their current place of residence for less than 3 years. Second, based on recent maternal migration status, disparities in full immunization coverage were observed across major states, among socio-economic groups, and for children of mothers with lower education levels. Third, children of recently migrated mothers had lower odds of receiving full immunization than children of non-migrated mothers, even after excluding data from children whose mothers were not living with their husbands, husbands with multiple wives, and visitors. Additionally, children whose mothers were recently migrated had lower odds of being fully immunized than both permanent residents and settled migrants. Fourth, a consistent finding was that children of recently migrated mothers, across household wealth quintiles (poorest, poor, middle, richer, and richest), had lower odds of achieving full immunization than their non-migrated counterparts. Fifth, similar disparities were observed across different social groups, with children of recently migrated mothers in these groups associated with lower odds of full immunization than children of non-migrated mothers.

Numerous studies have demonstrated that demographic, socio-economic, and geographic factors significantly influence immunization rates. However, there is limited evidence on the relationship between migration and childhood immunization in low-income countries, including India. Our study indicated that recent maternal migration of less than 3 years significantly contributes to lower immunization uptake among children aged 12–23 months in India. The analysis also highlights that recent maternal migration was consistently associated with lower odds of full immunization across household wealth status and social groups among children aged 12–23 months. The strength of the negative association varied across wealth and social groups, with the strongest association observed among the poorest households and the weakest among the richest households. Across social groups, the strongest association was observed among the Scheduled Caste children, whereas the weakest was observed among the Scheduled Tribe children. This pattern suggests that the association between recent maternal migration and lower odds of full immunization extends across different socioeconomic and social groups, although the strength of the association may vary by household wealth and social position. The existing studies support our findings, showing that children of recently migrated mothers are less likely to be fully immunized than natives and settled migrants [19,36]. These studies also define recent migration, similar to our study, as those who have lived at their current residence for at least 2 years. Kusuma et al. (2010, 2018) found that recent migrants (those who moved to the national capital, Delhi, from rural areas within the last 5 years) had a higher risk of incomplete immunization than settled migrants [18,28]. However, studies that did not consider recent migration, such as living in the current place for less than 3 years, were also found to be associated with lower routine immunization coverage among children. Antai D. (2010) and Kiros & White (2004) found that children of mothers who had migrated from rural to urban areas were less likely to be fully immunized than children of non-migrant mothers residing in rural areas, even after adjusting for several child, family, and village-level variables [23,37]. In addition, evidence from systematic reviews and meta-analyses also suggests that migrant children, especially recent migrants, are less likely to receive full immunization than non-migrant children [26,38]. 

Our results demonstrate how recent migration may influence children’s immunization uptake through the health care access livelihood framework and the theories of migrant disruption, migrant adaptation, and assimilation. Relocating to a current residential place, even within the same country or state, alters both social and physical environments, profoundly impacting health behaviors and outcomes. For recent migrants, whether moving from rural to urban areas or vice versa, their access to healthcare services is often restricted. This difficulty stems from a combination of factors in the host community. These factors include their living and working conditions, limited employment opportunities, inadequate social security benefits, and their social group dynamics. Collectively, these issues compromise their ability to afford essential healthcare services [14]. Additional specific barriers, including linguistic challenges, prevailing social norms, or cultural perceptions of health, may further restrict migrants’ access to services. Consistent with both our study and the theory of migrant adaptation, Azad and Islam’s study indicates that recent migrants have lower immunization rates than settled migrants [39]. This difference is likely due to an increased familiarity with local cultural practices, norms, and living conditions among settled migrants. In contrast, recent migrants remain more vulnerable, often lacking social networks, which may hinder their access. Poor adaptation also partly explains why migrant children have lower immunization uptake than urban non-migrant children. According to a regional study conducted in India [19], migrant parents’ use of immunization services is negatively affected by their limited knowledge of vaccination schedules and timing, as well as limited access to healthcare services. It is also evident that migrant children’s vaccination cards are often misplaced during migration, discouraging parents from immunizing their children and leading to interruptions in vaccination schedules. In addition to the disruption theory, our study also assumes that women often return to their native places (particularly in India and other South Asian countries) during childbirth and the immediate postnatal period for better care [40,41]. Studies from Indian states showed that one to two-thirds of women returned to their natal homes at some point during pregnancy, for childbirth, or postpartum, which can also lead to delays or under-immunization. 

Migrant individuals and families are considered high-risk groups due to their inherent vulnerabilities, which often result in reduced immunization rates and lower uptake of essential health care services. Structural barriers in healthcare systems disproportionately impede the access to, affordability of, and awareness of essential health care services, including immunization. These include challenges such as migrants’ long and inconvenient working hours coinciding with immunization session timings and the risk of losing daily wages, often referred to as the opportunity cost. In addition, migrants often face a lack of culturally and linguistically appropriate health information as well as health providers. Together, these challenges translate into an inability to prioritize healthcare over survival, often leading to delays in service uptake and worsening health conditions. Therefore, continuous efforts are required to ensure these populations are effectively reached and monitored. With rapidly growing urbanization in India, the projected proportions of urban and rural populations are expected to change rapidly over the next two decades. While the current urbanization rate is approximately 35%, it is projected to reach 46.5% by 2050 [42]. The primary factor contributing to this growing urban population is migration, which consequently strains health systems’ infrastructure and service delivery.

To effectively address these challenges, there is a clear need for a robust, inclusive tracking platform built around a unique, portable identifier, similar to a mobile number, that can track the child regardless of geographic movement. Such a system would enable both caregivers and frontline health workers to access and update vaccination records in real time, ensuring continuity of care even when families migrate. Evidence from multiple studies suggests that the use of unique digital identifiers for tracking migratory children significantly improves immunization coverage by reducing dropouts and missed doses.

In India, nearly 20% of children remain partially immunized, representing a critical “low-hanging fruit” for programmatic intervention, and a substantial proportion of these children belong to migrant populations. Additionally, zero-dose children, who account for roughly 4% of the cohort, also disproportionately come from migratory backgrounds. Frequent movement during early childhood disrupts immunization schedules, often compounded by limited awareness and weak linkages to local health systems. Addressing this gap requires a robust beneficiary-tracking mechanism that transcends administrative boundaries. A unified digital system with a unique ID can ensure that children are identified, tracked, and vaccinated regardless of their previous place of residence. This approach would not only facilitate timely follow-up for missed doses but also strengthen accountability within the system, thereby improving coverage and advancing the IA2030 goal.

The introduction of digital tools has further strengthened India’s capacity to track migratory and dropout cases effectively. U-WIN, a digital platform for vaccine registration, record-keeping, and tracking, built on the successful foundation of the CoWIN platform, enables real-time registration, tracking, and follow-up of beneficiaries across multiple locations. It is critical in tracing migratory children, ensuring scheduled dose completion, and tracking beneficiaries. It enables appointment booking, real-time data capture, certificate generation, and follow-up reminders to ensure complete vaccination. This strategy has strengthened the continuum of care. In conclusion, these mechanisms have the potential to address challenges in vaccine uptake and ensure that no child is left behind. 

### Limitations

Although this study reveals important relationships between migration status and FIC, it is limited by data unavailability. The current data is limited and does not include crucial information on the migratory patterns, specifically rural-to-urban and urban-to-rural movements. Additionally, the effect of parental migration (within and across states) on their children’s immunization status is not addressed. It lacks specific information on where migrants reside within urban settings, such as in slums or at their workplaces. Furthermore, NFHS-5 does not provide information on the reasons for migration or the origins of previous movements to current residences. Consequently, the survey was not designed to differentiate between specific migration timing—such as pre-pregnancy migration, temporary movement for childbirth, or migration occurring at various stages of the child’s immunization schedule. However, NFHS-5 does not contain direct measures of livelihood insecurity, unmet healthcare needs, health beliefs, social norms, access, affordability, awareness, or acceptance. The survey does not collect social network details for migrant groups, reasons for delays, or non-utilization of healthcare services. These limitations may affect the study’s outcomes.

## 5. Conclusions

The findings of this study provide quantitative evidence that recent migration (less than 3 years) of mothers is significantly associated with lower coverage of full immunization among children aged 12–23 months in India. Additionally, recent migration was consistently associated with lower odds of full immunization across household wealth levels and social groups. This study suggests that children of recently migrated women are a vulnerable subgroup at risk of not receiving full immunization. This study recommends that policymakers and health service administrators, especially in Maternal and Child Health, aim to include children of recently migrated women. Nevertheless, additional research on migration and childhood vaccination is needed to better understand mechanisms to enhance immunization coverage and uptake of health services among children.

## Figures and Tables

**Table 1:** Characteristics of the indicators, India, 2019–2021.

Background Characteristic	%, [95%, CI]	Unweighted Sample(n)
**Immunization Status**		
Under immunization	23.4, [22.8,24.0]	10,093
Full immunization	76.6, [76.0,77.2]	33,039
**Recent migration (<3 years)**		
No	90.4, [90.0,90.8]	39,486
Yes	9.6, [9.2,10.0]	3646
**Gender**		
Male	51.9, [51.3,52.6]	22,378
Female	48.1, [47.4,48.7]	20,754
**Mother’s age at birth**		
15–19 years	11.3, [10.9,11.7]	4437
20–29 years	74.5, [73.9,75.0]	31,772
30+years	14.3, [13.8,14.8]	6923
**Mother’s education in years**		
No schooling	19.1, [18.6,19.6]	8460
<5 years complete	4.4, [4.1,4.6]	2129
5–8 years complete	24.5, [24.0,25.1]	10,763
9–12 years	34.7, [34.1,35.4]	15,241
>12 years	17.3, [16.7,17.9]	6539
**Media Exposure**		
No	49.2, [48.6,49.9]	22,223
Yes	50.8, [50.1,51.4]	20,909
**Birth Order**		
1st order	39.5, [38.9,40.1]	16,753
2&3rd order	49.4, [48.7,50.0]	21,020
4th& more order	11.1, [10.7,11.6]	5359
**Institutional delivery**		
Public Health Facility	63, [62.3,63.7]	28,406
Private Health Facility	27.5, [26.9,28.2]	9723
No Health Facility	9.5, [9.1,9.9]	5003
**Intended Birth**		
Yes	92.3, [91.9,92.6]	39,994
No	7.7, [7.4,8.1]	3138
**Current place of residence**		
Urban	27, [26.4,27.5]	8817
Rural	73, [72.5,73.6]	34,315
**Social Groups**		
Schedule caste	23.1, [22.5,23.7]	8881
Schedule tribe	10, [9.6,10.4]	8495
OBC	43.4, [42.7,44.1]	16,603
Others	23.5, [22.9,24.2]	9153
**Wealth quintile**		
Poorest	23.8, [23.2,24.4]	11,289
Poorer	21.3, [20.8,21.8]	9988
Middle	19.9, [19.4,20.5]	8468
Richer	18.8, [18.2,19.4]	7400
Richest	16.2, [15.7,16.7]	5987
**Religion**		
Hindu	79.6, [78.9,80.3]	31,986
Muslim	16.1, [15.4,16.8]	6052
Christian	2, [1.8,2.2]	3323
Sikh	1.3, [1.1,1.4]	765
Others	1.1, [0.9,1.3]	1006
**Regions of India**		
North	13, [12.7,13.3]	7828
Central	27.7, [27.2,28.1]	11,197
East	26.4, [25.9,26.9]	8483
Northeast	3.5, [3.4,3.6]	6141
West	12.4, [12.0,12.9]	3871
South	16.9, [16.5,17.4]	5612
**Total**	**100**	**43** **,** **132**

Note: Percentages and 95% confidence intervals (CIs) are survey-weighted estimates accounting for the sampling design, while sample counts (n) are unweighted observations.

**Table 2:** Multivariate logistic regression on Full immunization among children 12–23 months, India, 2019–2021.

	Model-I	Model-II ^#^	Model-III ^##^
Background Characteristic	aOR, [95%, CI]	aOR, [95%, CI]	aOR, [95%, CI]
**Recent Migration (<3 years)**			
No ^Ref^	1	1	1
Yes	0.39 *** [0.35–0.43]	0.38 *** [0.34–0.44]	0.52 *** [0.45–0.62]
**Gender**			
Male ^Ref^	1	1	1
Female	0.96 [0.90–1.03]	0.96 [0.89–1.03]	0.97 [0.90–1.04]
**Mother’s age at birth**			
15–19 years ^Ref^	1	1	1
20–29 years	1.17 ** [1.05–1.31]	1.15 * [1.01–1.30]	1.14 * [1.00–1.30]
30+ years	1.29 ** [1.10–1.50]	1.24 * [1.05–1.47]	1.24 * [1.04–1.47]
**Mother’s education in years**			
No schooling ^Ref^	1	1	1
<5 years complete	1.45 *** [1.24–1.69]	1.44 *** [1.22–1.71]	1.48 *** [1.25–1.76]
5–8 years complete	1.40 *** [1.27–1.53]	1.41 *** [1.27–1.56]	1.42 *** [1.28–1.58]
9–12 years	1.50 *** [1.36–1.66]	1.51 *** [1.36–1.69]	1.52 *** [1.36–1.70]
>12 years	1.30 *** [1.13–1.49]	1.26 ** [1.08–1.47]	1.26 ** [1.07–1.48]
**Media Exposure**			
No ^Ref^	1	1	1
Yes	1.18 *** [1.09–1.27]	1.17 *** [1.08–1.28]	1.19 *** [1.09–1.30]
**Birth Order**			
1st order ^Ref^	1	1	1
2 & 3rd order	0.81 *** [0.75–0.87]	0.80 *** [0.73–0.88]	0.80 *** [0.73–0.87]
4th & more order	0.69 *** [0.61–0.77]	0.68 *** [0.59–0.78]	0.67 *** [0.58–0.76]
**Intended Birth**			
Yes ^Ref^	1	1	1
No	0.91 [0.80–1.04]	0.88 [0.76–1.02]	0.89 [0.77–1.02]
**Institutional delivery**			
Public Health Facility ^Ref^	1	1	1
Private Health Facility	0.80 *** [0.73–0.87]	0.79 *** [0.72–0.86]	0.79 *** [0.72–0.87]
No Health Facility	0.63 *** [0.57–0.70]	0.62 *** [0.55–0.69]	0.61 *** [0.55–0.69]
**Current place of residence**			
Urban ^Ref^	1	1	1
Rural	1.25 *** [1.13–1.38]	1.28 *** [1.15–1.42]	1.30 *** [1.17–1.45]
**Social Groups**			
Schedule caste ^Ref^	1	1	1
Schedule tribe	1.09 [0.94–1.25]	1.07 [0.92–1.25]	1.06 [0.90–1.24]
OBC	1.03 [0.94–1.12]	1.04 [0.94–1.14]	1.05 [0.95–1.16]
Others	0.94 [0.85–1.05]	0.94 [0.83–1.05]	0.94 [0.83–1.06]
**Wealth quintile**			
Poorest ^Ref^	1	1	1
Poorer	1.14 ** [1.04–1.24]	1.10 [1.00–1.22]	1.10 [0.99–1.21]
Middle	1.34 *** [1.21–1.49]	1.32 *** [1.18–1.49]	1.30 *** [1.15–1.46]
Richer	1.35 *** [1.19–1.55]	1.35 *** [1.16–1.56]	1.29 *** [1.11–1.50]
Richest	1.38 *** [1.18–1.62]	1.43 *** [1.21–1.68]	1.38 *** [1.17–1.63]
**Religion**			
Hindu ^Ref^	1	1	1
Muslim	0.80 *** [0.73–0.89]	0.80 *** [0.71–0.89]	0.79 *** [0.71–0.89]
Christian	1.17 [0.95–1.45]	1.12 [0.89–1.41]	1.16 [0.93–1.45]
Sikh	0.85 [0.67–1.08]	0.84 [0.66–1.09]	0.83 [0.65–1.08]
Others	1.33 [0.88–2.02]	1.42 [0.94–2.15]	1.37 [0.91–2.06]
**Regions of India**			
North ^Ref^	1	1	1
Central	0.73 *** [0.66–0.80]	0.74 *** [0.66–0.82]	0.72 *** [0.65–0.81]
East	1.17 ** [1.04–1.31]	1.20 ** [1.06–1.36]	1.16 * [1.02–1.32]
Northeast	0.53 *** [0.46–0.60]	0.52 *** [0.45–0.59]	0.50 *** [0.43–0.57]
West	0.75 *** [0.64–0.87]	0.77 ** [0.66–0.90]	0.76 *** [0.65–0.89]
South	1.12 [0.98–1.27]	1.10 [0.96–1.26]	1.07 [0.92–1.23]
**Sample**	**43** **,** **132**	**37** **,** **172**	**36** **,** **357**

Note: ^Ref^ = Reference; *p* value * *p* < 0.05, ** *p* < 0.01, *** *p* < 0.001; # Restricted to children whose mothers were currently living with their husbands/partners and whose husbands/partners had no other wives. ## Restricted to the same criteria as Model-II and additionally excluded temporary visitors to the current place of residence.

**Table 3:** Multivariate logistic regression on full immunization among children 12–23 months by mother’s recent migration and permanent residences, India, 2019–2021.

	Model-I	Model-II ^#^
Background Characteristic	aOR, [95%, CI]	aOR, [95%, CI]
**Recent Migration (<3 years)**		
No ^Ref^	1	1
Yes	0.67 *** [0.54–0.83]	0.70 * [0.53–0.92]
**Gender**		
Male ^Ref^	1	1
Female	1.14 [0.94–1.38]	1.22 [0.96–1.54]
**Mother’s age at birth**		
15–19 years ^Ref^	1	1
20–29 years	1.42 * [1.07–1.88]	1.29 [0.90–1.86]
30+ years	1.45 [0.95–2.22]	1.26 [0.75–2.11]
**Mother’s education in years**		
No schooling ^Ref^	1	1
<5 years complete	1.34 [0.85–2.12]	1.48 [0.85–2.58]
5–8 years complete	1.24 [0.91–1.68]	1.40 [0.96–2.02]
9–12 years	1.71 ** [1.24–2.36]	1.69 ** [1.14–2.51]
>12 years	1.30 [0.89–1.89]	1.27 [0.80–2.02]
**Media Exposure**		
No ^Ref^	1	1
Yes	1.03 [0.81–1.31]	1.07 [0.81–1.43]
**Birth Order**		
1st order ^Ref^	1	1
2 & 3rd order	0.77 * [0.61–0.96]	0.78 [0.59–1.03]
4th & more order	0.56 ** [0.37–0.86]	0.57 * [0.34–0.94]
**Intended Birth**		
Yes ^Ref^	1	1
No	1.03 [0.74–1.44]	1.06 [0.71–1.57]
**Institutional delivery**		
Public Health Facility ^Ref^	1	1
Private Health Facility	0.88 [0.69–1.12]	0.95 [0.71–1.28]
No Health Facility	0.59 ** [0.42–0.83]	0.49 *** [0.33–0.71]
**Current place of residence**		
Urban ^Ref^	1	1
Rural	0.91 [0.72–1.15]	0.94 [0.72–1.23]
**Social Groups**		
Schedule caste ^Ref^	1	1
Schedule tribe	1.29 [0.89–1.86]	1.38 [0.89–2.13]
OBC	1.09 [0.84–1.40]	1.15 [0.84–1.59]
Others	0.87 [0.62–1.21]	0.80 [0.53–1.21]
**Wealth quintile**		
Poorest ^Ref^	1	1
Poorer	1.41 * [1.06–1.87]	1.30 [0.91–1.87]
Middle	1.26 [0.91–1.75]	1.18 [0.79–1.75]
Richer	1.18 [0.82–1.70]	1.09 [0.70–1.69]
Richest	1.34 [0.86–2.09]	1.29 [0.76–2.17]
**Religion**		
Hindu ^Ref^	1	1
Muslim	1.18 [0.87–1.60]	1.37 [0.93–2.02]
Christian	1.47 [0.91–2.38]	1.62 [0.94–2.80]
Sikh	1.01 [0.49–2.08]	1.06 [0.44–2.57]
Others	1.94 [0.77–4.89]	6.00 *** [2.68–13.46]
**Regions of India**		
North ^Ref^	1	1
Central	0.69 * [0.50–0.96]	0.74 [0.50–1.10]
East	0.97 [0.69–1.37]	0.96 [0.63–1.47]
Northeast	0.48 ** [0.30–0.79]	0.40 *** [0.23–0.68]
West	0.73 [0.48–1.10]	0.76 [0.48–1.21]
South	1.32 [0.92–1.90]	1.28 [0.85–1.94]
**Sample**	**4763**	**3581**

Note: ^Ref^ = Reference; * *p* < 0.05, ** *p* < 0.01, *** *p* < 0.001; # Restricted to children whose mothers were currently living with their husbands/partners and whose husbands/partners had no other wives.

**Table 4:** Multivariate logistic regression on Full immunization among children 12–23 months by mother’s recent migration and settled migration, India, 2019–2021.

	Model-I	Model-II ^#^
Background Characteristic	aOR, [95%, CI]	aOR, [95%, CI]
**Recent Migration (<3 years)**		
No ^Ref^	1	1
Yes	0.52 *** [0.46–0.60]	0.56 *** [0.48–0.66]
**Gender**		
Male ^Ref^	1	1
Female	0.96 [0.90–1.03]	0.96 [0.89–1.03]
**Mother’s age at birth**		
15–19 years ^Ref^	1	1
20–29 years	1.19 ** [1.07–1.33]	1.18 * [1.04–1.34]
30+ years	1.33 *** [1.14–1.55]	1.29 ** [1.09–1.53]
**Mother’s education in the years**		
No schooling ^Ref^	1	1
<5 years complete	1.42 *** [1.22–1.65]	1.42 *** [1.20–1.68]
5–8 years complete	1.37 *** [1.25–1.50]	1.39 *** [1.25–1.53]
9–12 years	1.46 *** [1.32–1.61]	1.47 *** [1.32–1.64]
>12 years	1.23 ** [1.07–1.42]	1.21 * [1.04–1.41]
**Media Exposure**		
No ^Ref^	1	1
Yes	1.17 *** [1.09–1.26]	1.17 *** [1.07–1.27]
**Birth Order**		
1st order ^Ref^	1	1
2 & 3rd order	0.84 *** [0.77–0.91]	0.83 *** [0.76–0.90]
4th & more orders	0.73 *** [0.64–0.82]	0.71 *** [0.62–0.81]
**Intended Birth**		
Yes ^Ref^	1	1
No	0.89 [0.78–1.02]	0.86 * [0.73–1.00]
**Institutional delivery**		
Public Health Facility ^Ref^	1	1
Private Health Facility	0.79 *** [0.73–0.86]	0.79 *** [0.72–0.86]
No Health Facility	0.63 *** [0.57–0.70]	0.62 *** [0.55–0.69]
**Current place of residence**		
Urban ^Ref^	1	1
Rural	1.27 *** [1.15–1.41]	1.31 *** [1.17–1.46]
**Social Groups**		
Schedule caste ^Ref^	1	1
Schedule tribe	1.11 [0.96–1.28]	1.09 [0.93–1.27]
OBC	1.02 [0.94–1.11]	1.03 [0.94–1.14]
Others	0.95 [0.85–1.06]	0.94 [0.83–1.05]
**Wealth quintile**		
Poorest ^Ref^	1	1
Poorer	1.14 ** [1.05–1.25]	1.11 * [1.00–1.22]
Middle	1.37 *** [1.24–1.53]	1.35 *** [1.21–1.52]
Richer	1.40 *** [1.23–1.60]	1.38 *** [1.20–1.60]
Richest	1.47 *** [1.26–1.72]	1.50 *** [1.27–1.77]
**Religion**		
Hindu ^Ref^	1	1
Muslim	0.81 *** [0.73–0.89]	0.80 *** [0.71–0.89]
Christian	1.12 [0.91–1.39]	1.08 [0.86–1.36]
Sikh	0.87 [0.69–1.11]	0.86 [0.67–1.11]
Others	1.33 [0.88–2.01]	1.42 [0.95–2.13]
**Regions of India**		
North ^Ref^	1	1
Central	0.74 *** [0.67–0.82]	0.75 *** [0.68–0.84]
East	1.18 ** [1.06–1.32]	1.23 *** [1.09–1.39]
Northeast	0.57 *** [0.49–0.65]	0.55 *** [0.48–0.63]
West	0.77 *** [0.66–0.89]	0.78 ** [0.67–0.92]
South	1.13 [0.99–1.29]	1.09 [0.95–1.26]
**Sample**	**43** **,** **132**	**37** **,** **172**

Note: ^Ref^ = Reference; * *p* < 0.05, ** *p* < 0.01, *** *p* < 0.001; # Restricted to children whose mothers were currently living with their husbands/partners and whose husbands/partners had no other wives.

**Table 5:** Multivariate logistic regression on full immunization among children 12–23 months, stratified by household wealth status, India, 2019–2021.

	Poorest	Poor	Middle	Richer	Richest
Background Characteristic	aOR, [95%, CI]	aOR, [95%, CI]	aOR, [95%, CI]	aOR, [95%, CI]	aOR, [95%, CI]
**Recent Migration (<3 years)**					
No ^Ref^	1	1	1	1	1
Yes	0.29 *** [0.24–0.36]	0.43 *** [0.35–0.53]	0.34 *** [0.26–0.43]	0.42 *** [0.33–0.53]	0.52 *** [0.40–0.67]
**Gender**					
Male ^Ref^	1	1	1	1	1
Female	0.90 [0.81–1.01]	0.94 [0.83–1.06]	1.05 [0.91–1.21]	0.93 [0.78–1.11]	1.04 [0.87–1.25]
**Mother’s age at birth**					
15–19 years ^Ref^	1	1	1	1	1
20–29 years	1.26 * [1.04–1.52]	1.26 * [1.01–1.56]	1.00 [0.78–1.28]	1.28 [0.94–1.72]	0.78 [0.48–1.24]
30+years	1.39 ** [1.08–1.78]	2.06 *** [1.51–2.81]	1.28 [0.92–1.78]	0.97 [0.64–1.45]	0.79 [0.46–1.36]
**Mother’s education in years**					
No schooling ^Ref^	1	1	1	1	1
<5 years complete	1.52 *** [1.22–1.90]	1.50 ** [1.12–2.00]	1.47 [0.99–2.17]	1.22 [0.69–2.17]	1.18 [0.42–3.33]
5–8 years complete	1.43 *** [1.25–1.64]	1.43 *** [1.21–1.70]	1.42 ** [1.12–1.81]	1.31 [0.95–1.80]	1.03 [0.62–1.73]
9–12 years	1.58 *** [1.34–1.87]	1.46 *** [1.22–1.75]	1.59 *** [1.25–2.02]	1.41 * [1.04–1.93]	1.05 [0.64–1.71]
>12 years	1.44 [0.95–2.21]	1.72 ** [1.24–2.37]	1.54 ** [1.14–2.08]	1.16 [0.81–1.66]	0.84 [0.51–1.38]
**Media Exposure**					
No ^Ref^	1	1	1	1	1
Yes	1.31 ** [1.10–1.55]	1.17 * [1.02–1.34]	1.24 ** [1.06–1.46]	1.04 [0.86–1.25]	1.14 [0.91–1.44]
**Birth Order**					
1st order ^Ref^	1	1	1	1	1
2 & 3rd order	0.78 ** [0.67–0.91]	0.81 ** [0.70–0.94]	0.80 ** [0.67–0.95]	0.90 [0.74–1.09]	0.77 ** [0.63–0.94]
4th & more order	0.69 *** [0.57–0.84]	0.64 ** [0.49–0.84]	0.58 *** [0.44–0.76]	0.73 [0.51–1.04]	0.60 * [0.38–0.96]
**Intended Birth**					
Yes ^Ref^	1	1	1	1	1
No	0.95 [0.79–1.15]	0.97 [0.77–1.22]	0.81 [0.55–1.18]	0.93 [0.68–1.27]	0.80 [0.58–1.11]
**Institutional delivery**					
Public Health Facility ^Ref^	1	1	1	1	1
Private Health Facility	0.67 *** [0.54–0.81]	0.85 [0.72–1.01]	0.80 * [0.67–0.95]	0.85 [0.70–1.03]	0.85 [0.70–1.04]
No Health Facility	0.63 *** [0.55–0.72]	0.59 *** [0.49–0.72]	0.62 *** [0.48–0.80]	0.81 [0.53–1.24]	0.51 * [0.30–0.86]
**Current place of residence**					
Urban ^Ref^	1	1	1	1	1
Rural	1.54 ** [1.14–2.10]	1.42 ** [1.13–1.78]	1.30 ** [1.07–1.56]	1.04 [0.87–1.26]	1.21 * [1.02–1.44]
**Social Groups**					
Schedule caste ^Ref^	1	1	1	1	1
Schedule tribe	1.13 [0.96–1.33]	1.27 * [1.01–1.60]	1.01 [0.74–1.39]	0.87 [0.40–1.86]	0.67 [0.38–1.18]
OBC	0.93 [0.80–1.07]	1.01 [0.86–1.18]	0.93 [0.77–1.13]	1.22 [0.97–1.54]	1.22 [0.94–1.59]
Others	0.98 [0.78–1.21]	1.08 [0.85–1.37]	0.88 [0.69–1.12]	0.99 [0.76–1.28]	0.93 [0.71–1.21]
**Religion**					
Hindu ^Ref^	1	1	1	1	1
Muslim	0.72 *** [0.59–0.87]	0.94 [0.76–1.14]	0.82 [0.67–1.01]	0.67 *** [0.53–0.84]	0.81 [0.62–1.06]
Christian	0.81 [0.60–1.09]	0.77 [0.51–1.18]	1.37 [0.81–2.35]	1.42 [0.77–2.62]	2.25 ** [1.23–4.12]
Sikh	0.33 [0.07–1.52]	0.92 [0.46–1.86]	0.67 [0.40–1.15]	0.83 [0.55–1.25]	0.92 [0.65–1.29]
Others	1.56 * [1.01–2.42]	0.94 [0.46–1.93]	1.97 [0.83–4.70]	1.80 [0.66–4.91]	0.94 [0.34–2.60]
**Regions of India**					
North ^Ref^	1	1	1	1	1
Central	0.71 ** [0.57–0.89]	0.74 ** [0.60–0.91]	0.81 * [0.66–0.99]	0.72 ** [0.58–0.91]	0.64 *** [0.52–0.79]
East	1.14 [0.91–1.43]	1.41 ** [1.12–1.78]	1.36 * [1.06–1.73]	0.92 [0.68–1.24]	0.74 [0.55–1.01]
Northeast	0.57 *** [0.44–0.74]	0.49 *** [0.38–0.64]	0.54 *** [0.39–0.75]	0.52 ** [0.34–0.81]	0.53 * [0.30–0.94]
West	0.85 [0.61–1.19]	0.64 ** [0.47–0.87]	0.75 [0.56–1.01]	0.69 * [0.51–0.94]	0.89 [0.64–1.23]
South	0.89 [0.57–1.41]	1.23 [0.92–1.63]	1.20 [0.93–1.56]	1.18 [0.92–1.51]	0.84 [0.64–1.10]
**Sample**	**11** **,** **289**	**9988**	**8468**	**7400**	**5987**

Note: ^Ref^ = Reference; * *p* < 0.05, ** *p* < 0.01, *** *p* < 0.001.

**Table 6:** Multivariate logistic regression on full immunization among children 12–23 months, stratified by social groups, India, 2019–2021.

Background Characteristic	Schedule Caste	Schedule Tribe	OBC	Others
Recent Migration (<3 Years)	aOR, [95%, CI]	aOR, [95%, CI]	aOR, [95%, CI]	aOR, [95%, CI]
No ^Ref^	1	1	1	1
Yes	0.35 *** [0.28–0.42]	0.48 *** [0.34–0.69]	0.40 *** [0.35–0.46]	0.38 *** [0.30–0.48]
**Gender**				
Male ^Ref^	1	1	1	1
Female	0.86 * [0.76–0.99]	1.03 [0.86–1.24]	0.98 [0.89–1.07]	1.01 [0.88–1.17]
**Mother’s age at birth**				
15–19 years ^Ref^	1	1	1	1
20–29 years	1.38 ** [1.11–1.72]	1.13 [0.82–1.56]	1.08 [0.92–1.28]	1.12 [0.88–1.44]
30+years	2.02 *** [1.51–2.70]	1.08 [0.67–1.72]	1.10 [0.88–1.37]	1.18 [0.86–1.62]
**Mother’s education in years**				
No schooling ^Ref^	1	1	1	1
<5 years complete	1.50 * [1.08–2.09]	1.41 * [1.03–1.93]	1.28 * [1.00–1.64]	1.67 ** [1.16–2.40]
5–8 years complete	1.33 ** [1.12–1.58]	1.35 ** [1.08–1.70]	1.43 *** [1.24–1.64]	1.40 ** [1.12–1.76]
9–12 years	1.51 *** [1.24–1.82]	1.60 *** [1.25–2.05]	1.53 *** [1.32–1.77]	1.39 ** [1.09–1.76]
>12 years	1.22 [0.92–1.60]	0.70 [0.38–1.30]	1.33 ** [1.11–1.60]	1.38 * [1.03–1.83]
**Media Exposure**				
No ^Ref^	1	1	1	1
Yes	1.25 ** [1.08–1.46]	1.18 [0.96–1.46]	1.22 *** [1.09–1.37]	1.04 [0.89–1.22]
**Birth Order**				
1st order ^Ref^	1	1	1	1
2 & 3rd order	0.74 *** [0.63–0.87]	0.72** [0.58–0.90]	0.89 * [0.79–0.99]	0.80 * [0.68–0.95]
4th & more order	0.61 *** [0.48–0.78]	0.73* [0.55–0.97]	0.72 *** [0.60–0.86]	0.74 [0.54–1.01]
**Intended Birth**				
Yes ^Ref^	1	1	1	1
No	0.83 [0.64–1.07]	0.96 [0.69–1.33]	0.97 [0.81–1.17]	0.88 [0.69–1.12]
**Institutional delivery**				
Public Health Facility ^Ref^	1	1	1	1
Private Health Facility	0.81 * [0.68–0.97]	0.60 ** [0.42–0.86]	0.81 *** [0.72–0.91]	0.83 * [0.69–1.00]
No Health Facility	0.78 * [0.63–0.95]	0.46 *** [0.38–0.56]	0.63 *** [0.54–0.74]	0.60 *** [0.47–0.76]
**Current place of residence**				
Urban ^Ref^	1	1	1	1
Rural	1.40 ** [1.14–1.71]	1.08 [0.69–1.67]	1.09 [0.95–1.25]	1.45 *** [1.20–1.74]
**Wealth quintile**				
Poorest ^Ref^	1	1	1	1
Poorer	1.06 [0.89–1.27]	1.04 [0.84–1.29]	1.16 * [1.01–1.33]	1.41 ** [1.11–1.77]
Middle	1.43 ** [1.14–1.78]	1.27 [0.93–1.72]	1.33 *** [1.14–1.55]	1.58 *** [1.23–2.03]
Richer	1.33 * [1.03–1.72]	1.06 [0.62–1.81]	1.46 *** [1.22–1.75]	1.59 ** [1.19–2.13]
Richest	1.41 * [1.02–1.95]	1.11 [0.59–2.10]	1.42 ** [1.15–1.76]	1.68 ** [1.21–2.34]
**Religion**				
Hindu ^Ref^	1	1	1	1
Muslim	0.58 ** [0.40–0.82]	0.91 [0.50–1.64]	0.75 *** [0.66–0.85]	0.90 [0.76–1.07]
Christian	1.00 [0.59–1.69]	1.02 [0.72–1.43]	2.34 * [1.12–4.88]	2.19 * [1.10–4.36]
Sikh	0.57 ** [0.40–0.82]	0.55 [0.10–2.92]	2.01* [1.09–3.72]	1.06 [0.73–1.55]
Others	1.07 [0.55–2.11]	1.88 ** [1.24–2.84]	0.42 [0.12–1.46]	3.64 ** [1.64–8.06]
**Regions of India**				
North ^Ref^	1	1	1	1
Central	0.75 ** [0.61–0.92]	0.67 ** [0.50–0.89]	0.78 ** [0.67–0.91]	0.63 *** [0.51–0.76]
East	1.11 [0.88–1.39]	1.17 [0.86–1.60]	1.16 [0.97–1.38]	1.28* [1.02–1.61]
Northeast	0.45 *** [0.31–0.67]	0.43 *** [0.30–0.62]	0.67 ** [0.51–0.88]	0.53 *** [0.41–0.67]
West	0.59 ** [0.41–0.84]	0.83 [0.56–1.23]	0.87 [0.70–1.08]	0.69 ** [0.53–0.91]
South	1.31 * [1.00–1.72]	0.73 [0.48–1.11]	1.17 [0.97–1.41]	0.80 [0.60–1.06]
**Sample**	**8881**	**8495**	**16** **,** **603**	**9153**

Note: ^Ref^ = Reference; * *p* < 0.05, ** *p* < 0.01, *** *p* < 0.001.

## Data Availability

Data are available in a public, open-access repository. The data are available in the public domain and can be downloaded on reasonable request. https://dhsprogram.com/data/available-datasets.cfm (accessed on 9 March 2024).
